# Alternation between toxic and proliferative effects of Roundup® on human thyroid cells at different concentrations

**DOI:** 10.3389/fendo.2022.904437

**Published:** 2022-07-29

**Authors:** Izabela Fernanda Dal’ Bó, Elisângela Souza Teixeira, Larissa Teodoro Rabi, Karina Colombera Peres, Matheus Nascimento, Maria Izabel Chiamolera, Valdemar Máximo, Natássia Elena Bufalo, Laura Sterian Ward

**Affiliations:** ^1^ Laboratory of Cancer Molecular Genetics, School of Medical Sciences, University of Campinas, Campinas, Brazil; ^2^ São Leopoldo Mandic School of Medicine, Campinas, Brazil; ^3^ Department of Medicine, Federal University of São Paulo, São Paulo, Brazil; ^4^ Institute for Research and Innovation in Health (i3S), University of Porto, Porto, Portugal; ^5^ Institute of Molecular Pathology and Immunology of the University of Porto (Ipatimup), Porto, Portugal; ^6^ Department of Pathology, Faculty of Medicine of the University of Porto (FMUP), Porto, Portugal

**Keywords:** thyroid, endocrine disruptor, glyphosate, pesticide, cytotoxicity, proliferation

## Abstract

Endocrine-disrupting and carcinogenic effects of glyphosate have long been suspected, but little is known about the effect of compounds used in real life at different concentrations, neither in normal nor in thyroid tumor cells. As cancer cells may have different sensitivities and the effect of the product containing glyphosate may be different from that produced by the active ingredient alone, including the Acceptable Occupational Exposure Level (AOEL=160µg/L) and the Acceptable Daily Intake (ADI=830µg/L) determined by ANVISA, we used two human thyroid-derived cell lines, Nthy-ori 3-1 (from normal follicular cells) and TPC-1 (from papillary carcinoma), to test 15 different concentrations of Roundup® Original DI. Trypan blue (TB), CCK-8 and BrdU assays were used to evaluate cytotoxicity, metabolic activity and proliferation with 24h and 48h exposures in technical and biological triplicates. TB showed an important toxic effect, especially after 24h of exposure, in both cell lines. The AOEL concentration caused the death of 43% and 50% of the Nthy-ori and TPC-1 cells, respectively, in 24 h, while ADI resulted in 35% and 58% of cell death. After 48h of exposure, AOEL and ADI caused a lower number of dead Nthy-ori (33% and 18%) and TPC-1 (33% and 37%) cells, respectively, suggesting that the toxic effect of the product disappears and/or both strains have repair mechanisms that protect them from longer exposures. On the other hand, the CCK-8 assay showed that small concentrations of Roundup have a proliferative effect: 6.5µg/L increased the number of both Nthy-ori and TPC-1 cells at 24h, and the BrdU assay confirmed the stimulatory effect with a 321% increase in the absorbance of Nthy-ori cells at 48h. The herbicide produced even more frequent increases in the BrdU absorbance of TPC-1 cells, mainly at 24h. We conclude that thyroid cells exposed to Roundup present a nonmonotonic dual dose–response curve. Low concentrations of the pesticide, considered acceptable, cause significant cell death but also have an important proliferative effect, especially on TPC-1 cells. This herbicide, widely used around the world, may play a role in the increased incidence rate of thyroid nodules and cancer that has been observed in recent decades.

## Introduction

The incidence rate of thyroid cancer (TC) has increased dramatically in recent decades, making it one of the most frequent neoplasms and the fastest growing cancer in women almost all over the world (1). Although most of this increase is certainly due to the use of sensitive and accessible diagnostic methods such as cervical ultrasound, there is solid evidence that other factors may contribute. Indeed, large tumors have also been detected more frequently, and there have been major changes in the histological profile (2) and genetic landscape of currently diagnosed tumors (3).

Endocrine disruptors (EDs) have long been considered among those environmental factors most likely to be involved in thyroid tumorigenesis. These chemicals affect the endocrine system at the cellular and molecular levels (4, 5), and many EDs persist in the environment and become organic pollutants (6, 7). Because ED may be used in our routine in different environments, contact with low doses and exposure from a very early age, even in intrauterine life, may influence disease outbreaks, including thyroid diseases (4, 5).

ED impairs the action and activity of triiodothyronine (T3) and thyroxine (T4), which can interfere with several pathways of thyroid hormone metabolism (8). Furthermore, alterations in thyroid hormone homeostasis modify the hypothalamic-pituitary-thyroid axis, affecting thyrotrophic hormone (TRH) and thyroid stimulating hormone (TSH) (9, 10). Exposure to ED has been linked to hypothyroidism and the appearance of goiter (11). However, little is known about the concentrations at which chemical compounds have a disruptive effect on the thyroid and/or their mechanism of action.

Glyphosate-based herbicides have been widely used for over 4 decades thanks to the introduction of glyphosate-resistant genetically modified crops in most of the world’s major food producers (12). Glyphosate (N-(phosphonomethyl)glycine) is an organophosphate considered by the International Agency for Research on Cancer (IARC) to be a class 2A carcinogen (probably carcinogenic) (13), but although glyphosate-based herbicide toxic effects as endocrine disruptors have been demonstrated in several cell lines and experimental *in vivo* models, their effect in the real world, especially concerning humans, is still a matter of debate.

One of the main commercial glyphosate-based products is Roundup^®^, first patented by Monsanto. Glyphosate was classified as a low toxic product by the Brazilian National Health Surveillance Agency (ANVISA) (14), but the potential harmful effects of the various commercial formulations available as Roundup^®^, which contain various adjuvants added with the aim of improving the product’s competence, are still scarce (15). In fact, in certain formulations, the adjuvants are at higher concentrations than the active ingredient, and some of these adjuvants have been reported to cause different types of damage (16–20).

Key cellular processes, such as proliferation, cell mobility, apoptosis and differentiation, are regulated by hormones, including thyroid hormones, which have been clearly demonstrated to be affected by glyphosate (21). Therefore, an effect on thyroid cell proliferation and consequent goiter or even on thyroid malignancy is plausible. In addition, thyroid tumor cells could be more susceptible to the effects of the chemical (22). Furthermore, since the dose–response curve of ED is nonmonotonic, it is essential to evaluate the effects of different doses, including those considered safe for humans, such as the AOEL and ADI, available in Brazilian Technical Note (Process No. 25351.056754/2013-17) of ANVISA (23).

The present study was designed to verify the effects of different doses of Roundup^®^ Original DI in normal and papillary carcinoma thyroid lines.

## Materials and methods

### Cell culture

We used the Nthy-ori 3–1-cell line obtained from Sigma–Aldrich, St. Louis, USA (90011609) and the TPC-1-cell line, kindly provided by Prof. Dr. Valdemar de Jesus Conde Maximo, from the Institute of Pathology and Molecular Immunology - Ipatimup, University of Porto, Portugal. Both cell lines were grown in RPMI 1640 medium containing 10% fetal bovine serum, 1% penicillin–streptomycin and 250 mg/ml fungizone (Sigma–Aldrich, St. Louis, USA). Cells were kept at 37°C in a humidified environment with 5% CO_2_.

### Roundup® Original DI preparation

To expose thyroid cell lines to different concentrations of the herbicide from 6.5 µg/L to 6500 µg/L, in conditions as close as possible to real life such as in food cultures (24, 25); doses to which farm workers are exposed (26); levels found in food and in water (12, 27). We used Roundup® Original DI (Monsanto, São Paulo, Brazil), whose formulation contains 445 g/L (44.5% w/v (phosphonomethyl) glycine diammonium salt (glyphosate), 370 g/L (37.0% w/v) equivalent to N-(phosphonomethyl)glycine acid (glyphosate) and 751 g/L (75.1% w/v) other unspecified ingredients with a concentration of 1,566 g/L.

The herbicide was diluted in water, and treatment doses were prepared at different concentrations, including the doses referenced by ANVISA. We used the following formulas for the dilution (23, 28):


$$\frac{\begin{array}{c} Acceptable\;daily\;intake\\ 0,5\;mg/kg\;(ADI)\;x\;70\;kg\;\left(\text{average adult body weight}\right) \end{array}}{42\;L\;\left(\text{total body weight water}\right)}$$

$$\frac{\begin{array}{c} Acceptable\;level\;of\;occupational\;exposure\\ 0,1\;mg/kg\;(AOEL)\;x\;70\;kg\;\left(average\;adult\;body\;weight\right) \end{array}}{42\;L\;\left(\text{total body weight water}\right)}$$

### Trypan blue exclusion test

TPC-1 and Nthy-ori 3-1 cells were seeded at 3.7x10^4^ cells in 12-well culture plates and incubated for 24 hours for complete cell adhesion. After 24 hours, the cells were treated with Roundup^®^ Original DI ranging from 6.5 µg/L to 6500 µg/L. Exposures lasted 24 and 48 hours. We used a 1:1 dilution (10 µl cell suspension of each concentration in 10 µl Trypan Blue 0.4%) (Sigma–Aldrich, St. Louis, USA) for cell counting, which was performed with Countess^®^ II FL (Thermo Fisher Scientific). The results are expressed as the percentage of viable cells compared to controls. All tests were performed in triplicate. We used the trypan blue (TB) assay to choose the concentrations used in the following cytotoxicity assay.

### Cell counting kit – 8 test

Cytotoxicity was detected by CCK-8 assay, performed according to the Sigma–Aldrich protocol (St. Louis, USA). A total of 100 µl of cell suspension (5000 cells/well) of the TPC-1 and Nthy-ori 3-1 strains was dispensed in 96-well plates and incubated for 24 hours in a humidified incubator at 37°C and 5% CO_2_ for complete cell adhesion. After 24 hours, Roundup^®^ Original DI different dilutions were added: 6.5 µg/L; 65 µg/L; 160 µg/L (AOEL); 830 µg/L (ADI); 6500 µg/L upon trypan blue pretest selection. Cells were incubated for 24 and 48 hours. After that, 10 µl of CCK-8 solution (Sigma–Aldrich, St. Louis, USA) was added to each well of the plate and incubated for 3 hours in a CO2 incubator. Then, the absorbance or optical density (OD) at 450 nm was measured using a microplate reader (ELx808, Biotek, Winooski, VT, USA). The assay was performed in technical and biological triplicate, and the cellular sensitivity to the chemical was expressed as the percentage of viable cells compared to control cells using the following equation:


$$\text{Cell viability }\;\left(\%\right)=\frac{OD\;(Roundup)\;-OD\;\left(White\right)}{OD\;\left(Control\right)-OD\;\left(White\right)}x\;100$$

### BrdU cell proliferation assay

Cell proliferation was measured by the BrdU Cell Proliferation Assay Kit (Cell Signaling Technology®) according to the manufacturer’s instructions. A total of 100,000 cells/well of the TPC-1 and Nthy-ori 3-1 strains were dispensed in 96-well plates and incubated for 24 hours in a humidified incubator at 37°C and 5% CO2 for complete cell adhesion. Then, Roundup® Original DI treatments were added, and the lines were incubated at 24 and 48 hours of exposure. A 10X BrdU solution was added to the wells that were incubated for 2 hours. After incubation, 100 µl/well of the fixation/denaturation solution was added, and the plates were maintained at room temperature for 30 minutes. Then, 100 µl of 1X detection antibody solution was added and maintained at room temperature for 1 hour. After washing the plates 3 times with 1X Wash Buffer, 1X HRP-conjugated secondary antibody solution was added, and again, the plates were kept at room temperature for 30 minutes and washed 3 times with 1X Wash Buffer. After that, 100 µl of TMB substrate was added and incubated for 30 minutes at room temperature. At this stage, the color change of the plates was already observed; we added 100 µl of STOP solution to each well, and a microplate reader (ELx808, Biotek, Winooski, VT, USA) with absorbance at 450 nm was used to read the plates. The assay was performed in technical triplicate. Cell proliferation was expressed by absorbance and corrected with the blank result (control reaction).

### Statistical analysis

Statistical analysis was performed using the SAS System for Windows (Statistical Analysis System), version 9.4. SAS Institute Inc, 2002-2012, Cary, North Carolina, USA. In all analyzes non-parametric techniques were applied. To compare numerical measurements between concentration and its respective control, the Wilcoxon test was used for related samples.

To compare numerical measurements between concentrations, times and cell lines, ANOVA was used for repeated measurements with transformation by ranks. The significance level adopted for the statistical tests was 5%.

## Results

### Effect of Roundup® on cell viability

**Figure 1:**
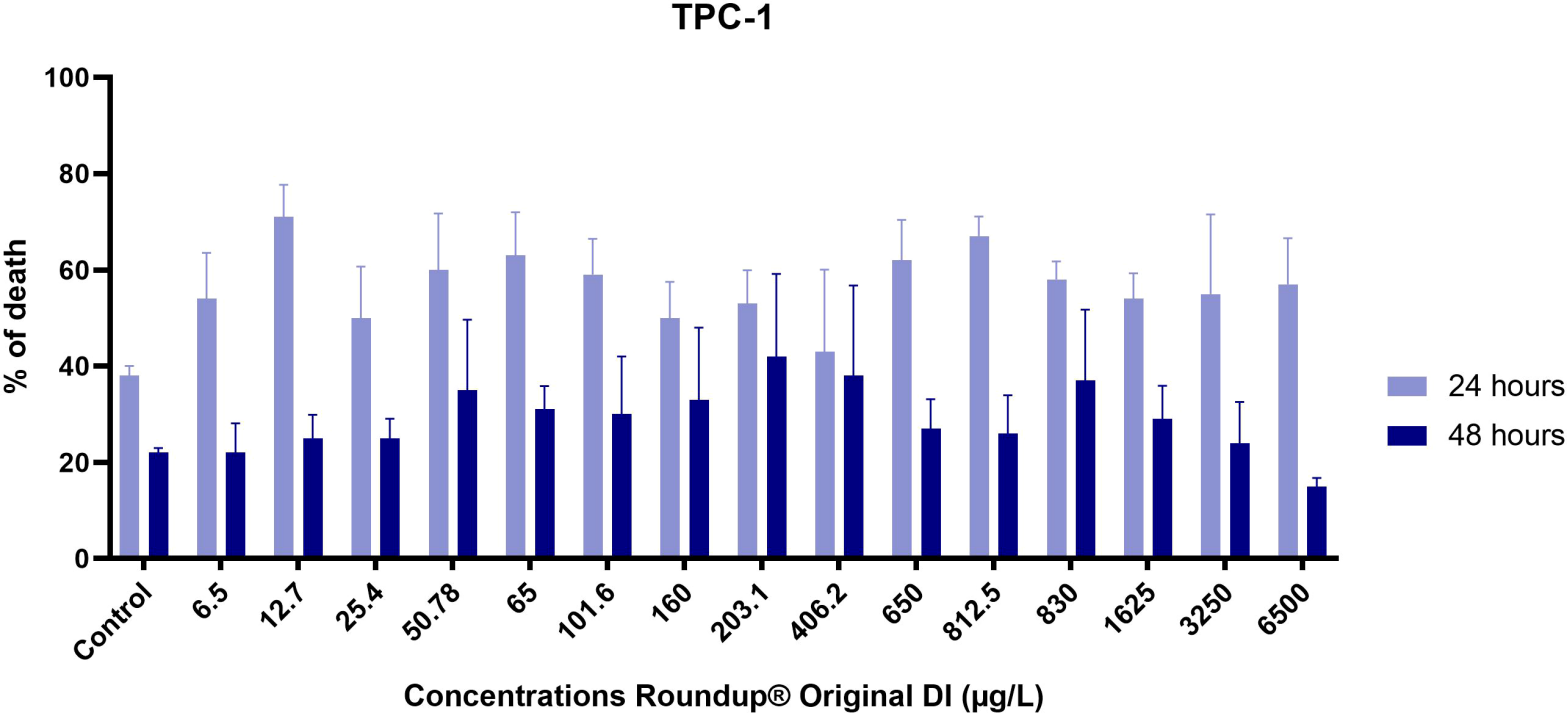
Percentage of dead TPC-1 cells after exposure to increasing concentrations of Roundup^®^ Original DI for 24 h and 48 h measured by the trypan blue dye test. Results are presented as mean ± SE.

We determined viability using loss of membrane integrity by trypan blue dye exclusion assay and, as demonstrated in **Figure 1**, observed that the effect of Roundup^®^ Original DI on TPC-1 thyroid cells was not dose-dependent but exhibited a nonmonotonic response within 24 h, which flattened out within 48 h.

Roundup® Original DI caused the death of up to 71% of TPC-1 cells exposed for 24 h at the tested concentrations, but this effect diminished after 48 h of exposure (**Figure 1**).

**Figure 2:**
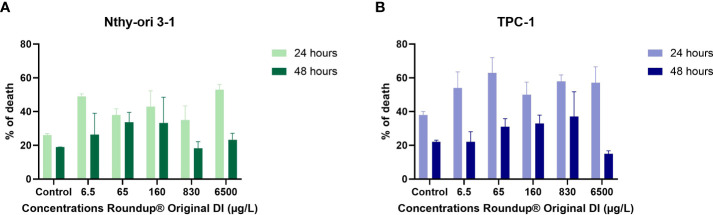
Comparison of cell mortality in Nthy-ori 3-1 **(A)** and TPC-1 **(B)** strains after exposure 216 to increasing concentrations of Roundup® Original DI for 24 h and 48 h by Trypan blue dye test.

We selected five concentrations of Roundup^®^ Original DI that were further applied to Nthy-ori 3-1 cells that **Figure 2A**, after 24 hours of exposure, also showed a decrease in viable cell numbers and, likewise TPC-1, became stable after 48 hours of exposure, as demonstrated in **Figure 2B** and **Table 1**.

**Table 1:** Percent mortality of Nthy-ori 3-1 and TPC-1 cells after exposure to Roundup® Original DI for 24 h and 48 h at different concentrations, measured by Trypan Blue exclusion test.

	24 hours				48 hours			
	Nthy-ori 3-1	p value	TPC-1	p value	Nthy-ori 3-1	p value	TPC-1	p value
6.5 μg/L	49% ± 1.5	0.5000	54% ± 9.5	0.5000	26% ± 12.6	1.0000	22% ± 6.0	0.5000
65 μg/L	38% ± 3.7	0.2500	63% ± 8.9	0.5000	34% ± 5.7	0.5000	31% ± 4.8	0.5000
160 μg/L	43% ± 9.2	0.5000	50% ± 7.5	1.0000	33% ± 15.1	0.5000	33% ± 4.9	0.2500
830 μg/L	35% ± 8.4	1.0000	58% ± 3.7	0.2500	18% ± 3.8	0.2500	37% ± 14.7	0.5000
6500 μg/L	53% ± 3.0	0.2500	57% ± 9.6	0.5000	23% ± 3.8	0.5000	15% ± 1.7	1.0000

Values are presented as mean ± SE. The p value refers to the comparison between the number of viable cells exposed to the herbicide and the number of viable control nonexposed cells.

We observed an important but limited cell injury rate, as detailed in **Table 1**. Exposure of Nthy-ori and TPC-1 cells to AOEL concentrations for 24 h caused the death of 43% and 50% of the cells, respectively, while ADI resulted in 35% and 58% cell death. After 48 h of exposure, AOEL and ADI caused a lower number of Nthy-ori (33% and 18%) and TPC-1 (30% and 37%) dead cells, respectively.

### Cytotoxicity assessment

**Figure 3:**
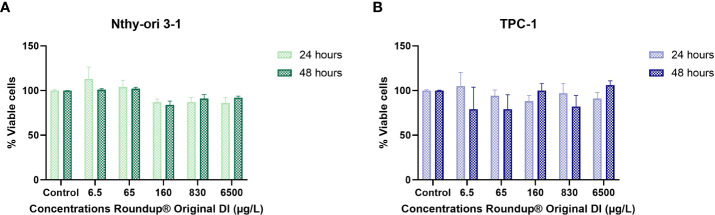
Comparison of cytotoxic effect on TPC-1 **(A)** and Nthy-ori 3-1 **(B)** strains after exposure to increasing concentrations of Roundup^®^ Original DI for 24 h and 48 h, measured by the CCK-8 assay and presented as mean percentage of viable cells ± SE.

To further investigate the product cytotoxicity, we performed a CCK-8 assay in normal and thyroid cancer cells after 24 h and 48 h exposure to Roundup^®^ Original DI at concentrations ranging from 6.5 μg/L to 6500 μg/L. **Figure 3** shows that both TPC-1 and Nthy-ori 3-1 cell viability was maintained above 79% at all tested concentrations.

In fact, the impact on cell viability was relatively small in both thyroid lineages. On the other hand, we observed that treatment with Roundup® Original DI produced proliferation of Nthy-ori 3-1 and TPC-1 cells at certain concentrations at both 24h and 48h.

**Figure 4:**
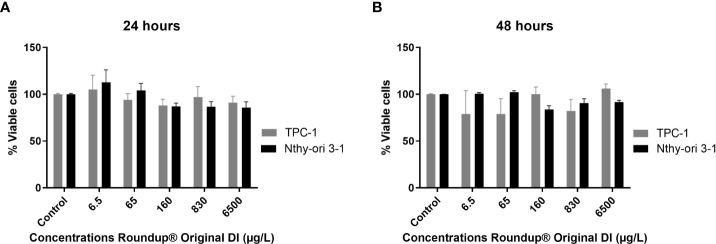
Comparison between the percentage of viable TPC-1 and Nthy-ori 3-1 cells at 24 h **(A)** and 48 h **(B)** of exposure to different concentrations of Roundup® Original DI using the CCK-8 assay and presented as mean percentage of viable cells ± SE.

The number of viable cells at 24 h and 48 h of exposure to Roundup® Original DI at the same doses showed that the herbicide had similar effects on Nthy-ori 3-1 and TCP-1 cells (**Figure 4**; **Table 2**).

**Table 2:** Percentage of viable Nthy-ori 3-1 and TPC-1 cells after exposure to Roundup® Original DI for 24 h and 48 h at different concentrations, measured by CCK-8 assay.

	Nthy-ori 3-1		TPC-1	
	24 hours	48 hours	24 hours	48 hours
6.5 μg/L	113% ± 13.44	101% ± 1.20	105% ± 15.3	79% ± 24.8
65 μg/L	104% ± 7.55	102% ± 1.73	94% ± 6.6	79% ± 16.2
160 μg/L	87% ± 3.60	84% ± 4.18	88% ± 6.6	100% ± 7.83
830 μg/L	87% ± 5.36	91% ± 4.48	97% ± 11.1	82% ± 12.3
6500 μg/L	86% ± 6.23	92% ± 1.76	91% ± 6.9	106% ± 4.93

Values are presented as mean ± SE.

We used ANOVA comparison for repeated measures (concentrations and times) considering the following elements: the type of cells; the concentrations and exposure times; and the interactions between them. By specifying the results of the concentrations*cell interaction in the Nthy-ori 3-1 strain, we found differences in concentrations such that 65 µg/L presented values greater than 160, 830 and 6500 µg/L, and that 160 µg/L was less than 830 µg/L. A post-hoc (contrast profile test) was performed to identify this difference. However, in the TPC-1 strain, no difference was observed between the concentrations. Furthermore, there was no significant difference between cells (Nthy-ori 3-1 and TPC-1) at any concentration (**Supplementary Table 1**).

### Influence of Roundup® on cell proliferation

**Figure 5:**
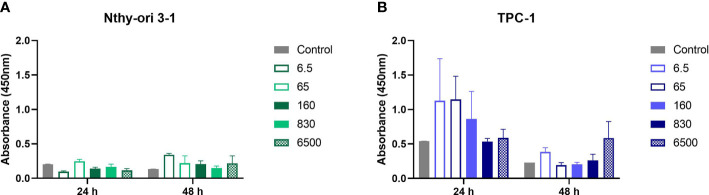
TPC-1 **(A)** and Nthy-ori 3-1 **(B)** cells were treated with increasing concentrations of Roundup® Original DI for 24 h and 48 h. Cell proliferation was measured by the BrdU absorbance, and readings were corrected by subtracting the blank results. Data are presented as the mean ± SE.

To further explore the proliferative effect of Roundup® Original DI, we employed a BrdU assay on both thyroid cell lines. As shown in **Figure 5**, BrdU incorporation confirmed the results of the CCK-8 assay, revealing that 24 h exposure to Roundup® Original DI increased the number of Nthy-ori 3-1 cells, especially at low concentrations. Roundup® had an even more important proliferative effect on TPC-1 cells, and all concentrations, except 830 µg/L, increased the number of cells at 24 h; the proliferative effect persisted after 48 h, except at 65 µg/L and 160 µg/L.

In fact, as shown in **Table 3**, 6.5 μg/L of the herbicide had an important toxic destructive effect on Nthy-ori 3-1 exposed for 24 h but also produced a striking proliferative effect after 48 h of exposure. TPC-1 cells were even more sensitive to the proliferative effect of Roundup®, which was observed at diverse concentrations, especially at 24 h of exposure and at a concentration of 6500 µg/L after exposure for 48 h.

**Table 3:** Absorbance of BrdU and percentage of Nthy-ori 3-1 and TPC-1 cells after exposure to Roundup® Original DI for 24 h and 48 h at different concentrations.

	Nthy-ori-3-1				TPC-1			
	24 hours		48 hours		24 hours		48 hours	
[]	ABS	%	ABS	%	ABS	%	ABS	%
Control	0.204 ± 0.0	100	0.134 ± 0.0	100	0.541 ± 0.0	100	0.229 ± 0.0	100
6.5 μg/L	0.097 ± 0.01	34	0.341 ± 0.02	321	1.128 ± 0.6	210	0.384 ± 0.6	208
65 μg/L	0.248 ± 0.03	124	0.220 ± 0.10	192	1.146 ± 0.34	214	0.193 ± 0.03	75
160 μg/L	0.140 ± 0.02	60	0.206 ± 0.05	178	0.865 ± 0.39	159	0.206 ± 0.03	85
830 μg/L	0.166 ± 0.04	75	0.147 ± 0.03	114	0.536 ± 0.04	96	0.264 ± 0.09	125
6500 μg/L	0.116 ± 0.02	46	0.218 ± 0.10	190	0.587 ± 0.13	106	0.586 ± 0.24	349

Values are presented as mean ± SE.

Comparing the results by ANOVA for repeated measures and using as factors the cell type; concentrations and exposure times; and the interactions between them, we found that the effect between cell types was significant, regardless of the exposure time and concentration. Evaluating the concentrations*times interaction that had a significant effect, at 48h the control was less than 6.5 ug/L, 6.5 ug/L was greater than 65, 160 and 830 ug/L and 65 ug/L was less than 6500 ug/L, in both cells. However, no significant differences were observed between the concentrations at 24 hours of exposure. The analysis of the times showed a significant difference just for one concentration (65 ug/L) that was greater at 24 h than at 48 h (**Supplementary Table 2**).

## Discussion

In the present study, we demonstrated that exposure to different doses of Roundup® Original DI, including the Acceptable Occupational Exposure Level (AOEL) and the Acceptable Daily Intake (ADI), according to Brazilian National Health Surveillance Agency (ANVISA), produce a nonmonotone dose–response curve on normal and mutated thyroid cells with alternation between toxic and proliferative effects at different doses. This effect is a well-known characteristic of endocrine disruptors (29). Roundup® Original DI produced a greater maximal reduction in membrane integrity of TPC-1 cells (63%) than Nthy-ori 3-1 with 49% cell death, but both cell lines seemed to recover after 48 h of exposure. Confirming previous reports, we also demonstrated that damage and mortality are not proportional to Roundup® concentrations (5, 6). In fact, thyroid cell mortality occurred even at low doses, such as those recommended in agriculture; to which the operator or worker may be exposed daily; and those corresponding to the maximum amount of a substance that can be ingested daily (12, 24–27). Conversely, other studies indicated a dose-dependent viability in human lymphocytes exposed to the pesticide (30). Chaufan et al found that a glyphosate formulation caused cytotoxicity in HepG2 cells depending on the dose and exposure time, even at dilutions below the recommended (31, 32). Another study, that used one of the formulated glyphosate-based products, also observed cytotoxic effects on HK-2 cells exposed for 24 hours at concentrations of 20-100 μM (equivalent to 3381.4-16907 µg/L), and the cell viability number was considerably reduced at concentrations above 40 μM (6762.8 µg/L). Furthermore, when the exposure times were extended to 36 and 48 hours, cell viabilities at concentrations of 40 and 60 μM (6762.8-10144.2 µg/L) were even more reduced (33). This difference could be related to a cell-specific sensitivity to the compound and to different effects of the active ingredient and commercially available formulations.

The important cell death rate observed in both normal and tumor thyroid cell lines exposed to low Roundup^®^ doses, including those considered acceptable by regulatory agencies, is relevant since the herbicide can be frequently found in the environment at these concentrations. In fact, water samples collected from irrigated rice field canals and weirs in Brazil had glyphosate concentrations of 144 µg/L (34). In Campeche, Mexico, glyphosate concentrations in groundwater were 1.42 μg/L and were 0.47 μg/L in urine samples from farmers (35). The compound has also been found in a wide range of concentrations varying from 0.15 ppm to 13 ppm (equivalent to 150-13000 µg/L) in many foods. Fruits, fresh vegetables and processed products ​​have 62 to 85 µg/L, a similar dose to the one we used in our experiments (36).

Interestingly, thyroid cells may adapt to the effects of the pesticide, since after 48 h, its harmful effect clearly diminishes. The activation of repair mechanisms may be responsible for damage control (31, 37). In fact, using *Anguilla anguilla* as an exposure model, Marques A et al showed that after the 7th and 14th days of exposure to Roundup®, DNA damage disappeared (38). Other mechanisms, including cell renewal, may justify the decrease in the number of cell deaths observed over time. Additionally, confirming our own data, a study involving the analysis of Roundup® toxicity in fish gill tissue demonstrated that the damage caused by the herbicide decreased as the exposure time increased (39). In addition, the effect of the pesticide may be tissue and/or cell specific. In fact, damage caused by Roundup^®^ Transorb in fish erythrocytes was greater after 24 and 96 hours than after 6 hours of exposure. However, in the gill cells, the results were different: at 96 h, there was not much damage, but great destruction was observed after 24 h of exposure (40). Another important issue is that different adjuvants added to the many commercially available glyphosate-derived herbicides can produce different effects, either potentiating the damage produced by glyphosate or, conversely, mitigating its effects. Benachour et al showed that cells derived from human embryos and human placentas manifested greater time-and dose-dependent cytotoxicity and a more potent effect of Roundup® than of the pure glyphosate compound (41).

Remarkably, Roundup Original DI^®^ also produced an increase in the viable cell rate, mainly in TPC-1 cells. This fact has been previously reported in human liver cancer (HepG2) cells after 4 hours of treatment at ADI and REL (residential exposure level) doses, equivalent to 2.91 μg/mL of the active ingredient (31).. Both glyphosate alone and some glyphosate-based herbicides, including Roundup®, have been reported to promote cell proliferation in breast cancer and adenocarcinoma cells (42). Lin and Garry reported 135% ± 3.5 and 126% ± 5.1 breast cancer cell proliferation rates with glyphosate and Roundup®, respectively (43). *In vitro* studies have also shown similar effects on HEC1A endometrial cancer cells (44). In fact, several studies have indicated that low concentrations of glyphosate and glyphosate-based herbicides effectively stimulate cell proliferation, especially in unstable and highly proliferating cells, such as tumor cells (43–46). Since TPC-1 cells are derived from papillary thyroid carcinoma, they harbor mutations, such as *RET/PTC* rearrangement, which confer greater resistance to apoptosis and cell death (47–51). This may explain the higher proliferation rate we observed in TPC-1 cells in comparison with the Nthy-ori 3-1-cell line, especially at 24 h (**Table 3**). Epidemiological data may be related, at least in part, with this proliferative effect. In fact, small thyroid carcinomas are frequently found in autopsies, and most of them are considered indolent (52). We hypothesize that exposure to endocrine disruptors contributes to the progression of these small lesions, contributing to the dramatic increase in thyroid cancer incidence rates, which parallels the increase in glyphosate-based herbicide usage rates.

It is worth remembering that these results are related to short-term exposure times to glyphosate, as in most studies involving this substance. In addition, the doses we used were limited and small compared to the broad spectrum that some regulatory agencies have established as tolerable for glyphosate in food and animal crops, ranging from 0 1 to 400 parts per million (or 100 to 400,000 ug/L) (53, 54). Also, it is possible that exposure to repeated doses of the chemical over a longer period may induce a cumulative effect with distinct consequences. A study carried out with rats exposed to 10 mg/kg glyphosate 3 times a week for 20 days observed a toxic effect of the chemical in liver tissue, which was able to induce cellular oxidative stress and activate apoptosis pathways (55). Another limitation to our study is the fact that we did not confirm our results with other similar assays, although the three assays we employed are robust and reliable. In addition, our data are specific to the cell types employed and may differ in other thyroid cells. They are also specific for the glyphosate-containing product tested (Roundup Original DI^®^) since other formulations have different adjuvants, which are not always well identified in the product leaflet.

The risk of cancer in humans upon use of glyphosate is still not conclusive, and although the IARC from the World Health Organization (WHO) established glyphosate as a probable human carcinogen (13), the European Food Safety Authority (EFSA) concluded that the herbicide does not prove to be carcinogenic or mutagenic (56). Our data highlight the importance of studying very carefully the implications of various dosages and co-formulants in the pesticide (57). In addition, further epidemiological evidence is urgently needed to evaluate the potential adverse effects of glyphosate products on sensitive human populations, particularly pregnant women, children and individuals with benign thyroid diseases.

Contribution to the field: Glyphosate-based herbicides, such as Roundup®, have environmental dispersion, and their accumulation can cause several effects on thyroid function, but their effects on normal and mutant thyroid cells are still poorly understood. This study shows that Roundup® Original DI acts on normal and thyroid papillary carcinoma cells at various concentrations, evidencing a dual toxic and proliferative effect. This herbicide, widely used around the world, may play a role in the increased incidence rate of thyroid nodules and cancer that has been observed in recent decades.

## Data availability statement

The original contributions presented in the study are included in the article/**Supplementary material**. Further inquiries can be directed to the corresponding author.

## Author contributions

All authors contributed to the concept and design of this study or to data acquisition and interpretation. All authors contributed to the review of the manuscript and read and approved the submitted version. All authors contributed to the article and approved the submitted version.

## Acknowledgments

We are grateful for the contributions of agronomists Guilherme Guimarães and Luis Carlos Castanheira for important discussions, clarifications and technical information and to the biostatistics services of the Faculty of Medical Sciences at UNICAMP. The authors are also grateful to the American Journal Experts for the linguistic services provided and to the Fundação de Amparo à Pesquisa do Estado de São Paulo (FAPESP), grant number 2020/02167-3; and to the Coordination for the Improvement of Higher Education Personnel (CAPES), grant number 88887.465269/2019-00, for the scholarship and financial support. LSW is a Category 1 Research Fellow at the National Council for Scientific and Technological Development (CNPq).

## Conflict of interest

LSW is a Category 1 Research Fellow at the National Council for Scientific and Technological Development (CNPq).

The remaining authors declare that the research was carried out in the absence of any commercial or financial relationship that could be interpreted as a potential conflict of interest.

## Publisher’s note

All claims expressed in this article are solely those of the authors and do not necessarily represent those of their affiliated organizations, or those of the publisher, the editors and the reviewers. Any product that may be evaluated in this article, or claim that may be made by its manufacturer, is not guaranteed or endorsed by the publisher.

## Abbreviations

ADI, Acceptable Daily Intake; AOEL, Acceptable Occupational Exposure Level; CCK-8, Cell Counting Kit – 8; ED, Endocrine disruptors; EFSA, European Food Safety Authority; GBH, Glyphosate-based herbicides; IARC, International Agency for Research on Cancer; ANVISA, National Health Surveillance Agency; OD, Optical density; REL, Residential exposure level; TC, Thyroid cancer; TRH, Thyrotrophic hormone; TSH, Thyroid stimulating hormone; T4, Thyroxine; T3, Triiodothyronine; TB, Trypan Blue; WHO, World Health Organization.
