# Supplementary material for: Alternation between toxic and proliferative effects of Roundup® on human thyroid cells at different concentrations
Source: Front Endocrinol (Lausanne). 2022 Jul 29;13:904437. doi: 10.3389/fendo.2022.904437 (PMC9382701; doi:10.3389/fendo.2022.904437)
Supplement: Supplementary file 1 [file Table_1.docx]

**Supplementary table 1.** ANOVA results for repeated measures compared by CCK-8 assay (% viability cells*) between cell types, concentrations and times (n=3).

| **Cells** | **Concentrations (µg/L)** | **Times** | **Interactions** | | | |
| --- | --- | --- | --- | --- | --- | --- |
| Nthy-ori 3-1 and TPC-1 | 6.5, 65, 160, 830, 6500 | 24h and 48h | Concentrations*cell | Times*cells | Concentrations*times | Concentrations*times*cell |
| 0.9296 | 0.0436 | 0.5615 | **0.0471^a^** | 0.9839 | 0.5633 | 0.5434 |

*Variable transformed into ranks for analysis.

^a^ Significant effect of concentration*cell interaction: significant differences between concentrations in Nthy-ori 3-1 cells (p=0.0013).

**Supplementary table 2.** ANOVA results for repeated measures comparing BrdU assay (cell proliferation absorbance*) between cell types, concentrations and times (n=3).

| **Cells** | **Concentrations (µg/L)** | **Times** | **Interactions** | | | |
| --- | --- | --- | --- | --- | --- | --- |
| Nthy-ori 3-1 and TPC-1 | Control, 6.5, 65, 160, 830, 6500 | 24 h and 48 h | Concentrations*cell | Times*cells | Concentrations*times | Concentrations*times*cell |
| **0.0005^a^** | 0.9155 | 0.2219 | 0.5103 | 0.0764 | **0.0011^b^** | 0.4448 |

*Variable transformed into ranks for analysis.

^a^ Significant effect for lineage type (TPC-1>Nthy-ori 3-1).

^b^ Significant effect of the concentration*time interaction: significant differences between concentrations at 48 h (p=0.0197). No differences were observed at 24 h (p=0.1068). Significant differences between times in control (p<0.0001, 24 h>48 h) and concentration 65 (p=0.0369, 24 h>48 h).
